# Clinical and Economic Consequences of Failure of Initial Antibiotic Therapy for Patients with Community-Onset Complicated Intra-Abdominal Infections

**DOI:** 10.1371/journal.pone.0119956

**Published:** 2015-04-24

**Authors:** Yong Pil Chong, In-Gyu Bae, Sang-Rok Lee, Jin-Won Chung, Jae-Bum Jun, Eun Ju Choo, Soo-youn Moon, Mi Suk Lee, Min Hyok Jeon, Eun Hee Song, Eun Jung Lee, Seong Yeon Park, Yang Soo Kim

**Affiliations:** 1 Department of Infectious Diseases, Asan Medical Center, University of Ulsan College of Medicine, Seoul, Republic of Korea; 2 Department of Internal Medicine, Gyeongsang National University School of Medicine, Jinju, Republic of Korea; 3 Department of Internal Medicine, CheongJu St. Mary's Hospital, CheongJu, Republic of Korea; 4 Department of Internal Medicine, Chung-Ang University College of Medicine, Seoul, Republic of Korea; 5 Department of Internal Medicine, Ulsan University Hospital, University of Ulsan College of Medicine, Ulsan, Republic of Korea; 6 Department of Internal Medicine, Soon Chun Hyang University Bucheon Hospital, Bucheon, Republic of Korea; 7 Department of Internal Medicine, Kyung Hee University School of Medicine, Seoul, Republic of Korea; 8 Department of Internal Medicine, Soon Chun Hyang University Cheonan Hospital, Cheonan, Republic of Korea; 9 Department of Internal Medicine, GangNeung Asan Hospital, University of Ulsan College of Medicine, GangNeung, Republic of Korea; 10 Department of Internal Medicine, Soon Chun Hyang University Seoul Hospital, Seoul, Republic of Korea; 11 Department of Internal Medicine, Dongguk University Ilsan Hospital, Ilsan, Republic of Korea; Vanderbilt University, UNITED STATES

## Abstract

**Objectives:**

Complicated intra-abdominal infection (cIAI) is infection that extends beyond the hollow viscus of origin into the peritoneal space, and is associated with either abscess formation or peritonitis. There are few studies that have assessed the actual costs and outcomes associated with failure of initial antibiotic therapy for cIAI. The aims of this study were to evaluate risk factors and impact on costs and outcomes of failure of initial antibiotic therapy for community-onset cIAI.

**Methods:**

A retrospective study was performed at eleven tertiary-care hospitals. Hospitalized adults with community-onset cIAI who underwent an appropriate source control procedure between August 2008 and September 2011 were included. Failure of initial antibiotic therapy was defined as a change of antibiotics due to a lack of improvement of the clinical symptoms and signs associated with cIAI in the first week.

**Results:**

A total of 514 patients hospitalized for community-onset cIAI were included in the analysis. The mean age of the patients was 53.3 ± 17.6 years, 72 patients (14%) had health care-associated infection, and 48 (9%) experienced failure of initial antibiotic therapy. Failure of initial antibiotic therapy was associated with increased costs and morbidity. After adjustment for covariates, patients with unsuccessful initial therapy received an additional 2.9 days of parenteral antibiotic therapy, were hospitalized for an additional 5.3 days, and incurred $3,287 in additional inpatient charges. Independent risk factors for failure of initial antibiotic therapy were health care-associated infection, solid cancer, and APACHE II ≥13.

**Conclusions:**

To improve outcomes and costs in patients with community-onset cIAI, rapid assessment of health care-associated risk factors and severity of disease, selection of an appropriate antibiotic regimen accordingly, and early infection source control should be performed.

## Introduction

Complicated intra-abdominal infection (cIAI) is an infection that extends beyond the hollow viscus of origin into the peritoneal space, causing peritonitis or abscess formation. These infections typically require both surgical or percutaneous intervention and intravenous antimicrobial therapy, and are associated with substantial morbidity and mortality. Despite improvements in the management of cIAIs, mortality rates are still high, ranging from 2% in appendicitis to as high as 30–35% in patients with peritonitis, depending on the anatomic origin, severity of infection, and comorbidity [1–3]. cIAIs include community-acquired infections, where the gastrointestinal perforation may be located in the stomach, duodenum, jejunum, ileum, appendix, or colon; and health care-associated infections, most commonly as a result of complications of elective or emergency abdominal operations. Community-onset infections include community-acquired infections and community-onset health care–associated infections [4].

Initial antibiotic therapy for cIAIs is usually empirical and sometimes problematic due to increasing antibiotic resistance. Whereas empirical therapy with broad-spectrum antibiotics might be more effective, its benefits must be weighed against its higher cost and the potential risk of increasing antibiotic resistance. In contrast, although empirical therapy with narrow-spectrum antibiotics may be less costly, it may be associated with a higher rate of clinical failure, thereby increasing total hospital costs. In addition to antibiotic administration, adequate source control is crucial for successful therapy of cIAIs. There are few studies that have assessed the actual costs and outcomes associated with failure of initial antibiotic therapy for cIAIs [5–8]. In these studies, the impact of health care-associated infection was not assessed. The main objectives of this study were to determine the frequency of failure of initial antibiotic therapy for community-onset cIAIs (including health care-associated cIAIs) and to determine the associated impact on medical costs and clinical outcomes. Secondary objectives were to identify potential risk factors contributing to failure of initial antibiotic therapy and to determine the epidemiology of the microorganisms in South Korea.

## Methods

### Ethics Statement

This study was reviewed and approved by the Institutional Review Boards of Asan Medical Center (IRB number: 2011–0719), Gyeongsang National University Hospital (2011–082), CheongJu St. Mary's Hospital (IRB-53), Chung-Ang University Hospital (C2011161), Kyung Hee University Hospital (1126–04), Soon Chun Hyang University Bucheon Hospital (2011–85), Ulsan Unversity Hospital (2011–092), Soon Chun Hyang University Cheonan Hospital (2011–105), GangNeung Asan Hospital (2011–038), Soon Chun Hyang University Seoul Hospital (2011–109), and Dongguk University Ilsan Hospital (2011–73). Written patient consent was waived by all sites in view of the retrospective and observational nature of the study. The complete protection of patients’ personal data was guaranteed according to the South Korea’s Bioethics and Safety Act.

### Study population and design

A retrospective study was performed at eleven tertiary-care hospitals in South Korea. Hospitalized adults (≥18 years old) with community-onset cIAI who underwent an appropriate source control procedure such as laparotomy, laparoscopy, or percutaneous drainage of an intra-abdominal abscess between August 2008 and September 2011 were included. cIAIs included conditions such as intra-abdominal abscess related to previous intra-abdominal operations, secondary bacterial peritonitis, appendicitis complicated by perforation and/or a periappendiceal abscess, perforated diverticulitis complicated by abscess formation or fecal contamination, complicated cholecystitis with evidence of perforation, empyema, or gangrene, perforation of a gastric or duodenal ulcer with symptoms exceeding 24 hours in duration, and perforation of the large or small intestine with abscess or fecal contamination [9]. Computerized patient records were used to identify potential study subjects using the International Classification of Disease 10^th^ edition codes. Then, manual reviews of their medical records were conducted to confirm whether study criteria were met.

Community-onset infection was classified into community-acquired infection and health care-associated infection. Community-onset health care–associated infection was defined when at least one of the following health care risk factors was present: [1] presence of an invasive device (eg, vascular catheter and gastric feeding tube) at the time of admission; [2] a history of methicillin-resistant *Staphylococcus aureus* (MRSA) infection or colonization; and [3] a history of surgery, hospitalization, dialysis, or residence in a long-term care facility in the 12 months preceding the diagnosis of cIAI [4,10].

### Data collection and definitions

Demographic characteristics, laboratory results, underlying diseases or conditions, site of infection, microbiology and antibiogram results, patient management (including source control and antimicrobial therapy received), clinical outcomes, and direct medical costs from the perspectives of the Korean National Health Insurance Corporation were recorded. Failure of initial antibiotic therapy (unsuccessful initial antibiotic therapy) was defined as a change of antibiotics due to a lack of improvement of the clinical symptoms and signs associated with cIAI within the first week of admission. A change to narrower spectrum antibiotics in patients with clinical improvement was not considered as failure of initial antibiotic therapy. To clearly compare patients with unsuccessful initial antibiotic therapy and those with successful initial antibiotic therapy, patients who underwent additional operative intervention or died within the first week of admission were excluded in the analysis.

### Outcomes

The primary outcomes were frequency of failure of initial antibiotic therapy, total length of hospital stay, total parenteral antibiotic days, overall medical costs, and in-hospital mortality. The overall medical costs of hospitalization were calculated using standardized reimbursement rates set by the Korean National Health Insurance System. This was possible because South Korea has a national health insurance system with universal coverage of the population. The secondary outcomes were the assessment of risk factors associated with failure of initial antibiotic therapy and the epidemiology of the microorganisms isolated in community-onset cIAIs.

### Statistical analysis

Comparisons between patients with unsuccessful initial antibiotic therapy and those with successful initial antibiotic therapy were performed. Categorical variables were compared using the χ^2^ test or Fisher’s exact test, as appropriate. Normally and non-normally distributed continuous variables were compared using Student’s *t*-test and the Mann-Whitney U test, respectively. Significant variables with P values less than 0.05 in the univariate analysis were included in the multiple logistic regression model to identify independent risk factors associated with failure of initial antibiotic therapy.

The Republic of Korea has universal health coverage of its population, and the national health insurance system pays health care providers on a fee-for-service basis. The overall costs of antibiotic therapy and hospitalization in patients with cIAI were calculated using the standardized reimbursement rates set by the Korean National Health Insurance System. To investigate the factors that affected parenteral antibiotic days, length of hospitalization, and overall costs, univariate linear regression analyses were carried out and the independent covariates were indentified through multiple linear regression analysis. Because the distribution of these variables was skewed, a natural log transformation was performed for the analysis. An analysis of covariance model was used to assess the adjusted mean differences in parenteral antibiotic days, hospital length of stay, and overall costs between the patient groups of successful initial therapy and unsuccessful initial therapy. A two-tailed P value less than 0.05 was considered statistically significant. All statistical analyses were performed using SPSS software, version 18.0 (SPSS Inc., Chicago, IL, USA).

## Results

### Baseline and clinical characteristics

A total of 539 consecutive patients hospitalized for community-onset cIAI were included. Of these patients, 25 patients who underwent additional operative intervention or died within the first week of admission were excluded and a total of 514 patients were finally included in the analysis. The mean age of the patients was 53.3 ± 17.6 years, 61% (312/514) were male, and 14% (72/514) had health care-associated infection. The most common intraabdominal diagnosis was complicated appendicitis (52%), followed by perforation of the intestine (17%), and perforated duodenal ulcer (16%). Half of the patients had generalized peritonitis. Among the patients included in the study, 180 had documented information on the pathogen (initial positive culture results). Baseline and clinical characteristics were similar between patients with positive culture and patients without positive culture.

Initial antibiotic therapy was successful in 466 (86%) patients and unsuccessful in 48 (9%) patients. Table 1 shows the baseline characteristics of these two groups. Patients with unsuccessful initial antibiotic therapy were more likely to be older, to have received ICU care, and to have health care-associated infection, underlying medical conditions, intestinal perforation, and severe disease than patients with successful initial antibiotic therapy. Time to the operation or intervention was similar between the two groups.

**Table 1:** Baseline and clinical characteristics of 514 patients with community-onset complicated intraabdominal infection stratified by failure or success of initial antibiotic therapy.

Characteristic	Total,	Initial antibiotic therapy		*P* value
	n = 514 (%)	Failure,	Success,	
		n = 48 (%)	n = 466 (%)	
Age, median (IQR)	53.5 (39–68)	60 (52–72.5)	53 (38–67)	0.004
Male	312 (60.7)	29 (60.4)	283 (60.7)	0.966
ICU care	165 (32.1)	23 (47.9)	142 (30.5)	0.014
Epidemiologic category				<0.001
Community-acquired	442 (86.0)	21 (43.8)	421(90.3)	
Health care-associated	72 (14.0)	27 (56.3)	45 (9.7)	
Underlying medical conditions				
None	282 (54.9)	17 (35.4)	265 (56.9)	0.004
Diabetes mellitus	48 (9.3)	5 (10.4)	43 (9.2)	0.794
Heart disease	21 (4.1)	5 (10.4)	16 (3.4)	0.037
Neurologic disease	28 (5.4)	7 (14.6)	21 (4.5)	0.010
Chronic renal failure	10 (1.9)	3 (6.3)	7 (1.5)	0.057
Liver cirrhosis	13 (2.5)	3 (6.3)	10 (2.1)	0.112
Hematologic malignancy	6 (1.2)	2 (4.2)	4 (0.9)	0.100
Solid tumor	29 (5.6)	9 (18.8)	20 (4.3)	0.001
Alcohol abuse	11 (2.1)	1 (2.1)	10 (2.1)	0.999
Steroid user	6 (1.2)	1 (2.1)	5 (1.1)	0.446
Primary intraabdominal diagnosis				
Intraabdominal abscess	13 (2.5)	2(4.2)	11(2.4)	0.347
Complicated appendicitis	268 (52.1)	16 (33.3)	252 (54.1)	0.006
Complicated diverticulitis	12 (2.3)	2 (4.2)	10 (2.1)	0.311
Complicated cholecystitis	13 (2.5)	2 (4.2)	11 (2.4)	0.347
Perforated gastric ulcer	33 (6.4)	6 (12.5)	27 (5.8)	0.111
Perforated duodenal ulcer	82 (16.0)	4 (8.3)	78 (16.7)	0.130
Perforation of intestine	86 (16.7)	16 (33.3)	70 (15.0)	0.001
Other	7 (1.3)	-	7 (1.5)	-
Anatomic site of infection^a^				
Appendix	273 (53.1)	16 (33.3)	257 (55.2)	0.004
Colon	72 (14.0)	12 (25.0)	60 (12.9)	0.021
Small bowel	37 (7.2)	6 (12.5)	31 (6.7)	0.142
Stomach/duodenum	120 (23.3)	10 (20.8)	110 (23.6)	0.666
Other	28 (5.4)	5 (10.4)	23 (4.9)	0.168
Infectious process				
Generalized peritonitis	254 (49.4)	29 (60.4)	225 (48.3)	0.109
Localized infection	229 (44.6)	17 (35.4)	212 (45.5)	0.181
Multiple abscesses	5 (1.0)	1 (2.1)	4 (0.9)	0.389
Single abscess	26 (5.1)	1 (2.1)	25 (5.4)	0.497
APACHE II ≥13	110 (21.4)	18 (37.5)	92 (19.7)	0.004
Time to first operation or intervention, h, mean (IQR)	7 (4.5–12)	8 (4.7–22)	7 (4.5–12)	0.269

Except where noted, values in parentheses indicate percentages.

IQR, interquartile range; APACHE, Acute Physiology and Chronic Heath Evaluation.

^a^ Some patients had multiple sites of infection.

### Pathogens

Bacterial organisms isolated from cultures of blood and surgical samples obtained during or within 48 hours of surgery/intervention are shown in Table 2. There were 223 pathogens isolated from 180 patients. Two or more pathogens were isolated in 39 patients. *Escherichia coli* was the most frequently identified pathogen (42%), followed by streptococcus species (15%), *Enterococcus* species (12%), and *Klebsiella pneumoniae* (8%). When compared between patients with unsuccessful and successful initial antibiotic therapy, there was no significant difference in the distribution of pathogens and antibiotic susceptibility patterns. *Streptococcus* species were marginally more likely to be isolated in community-acquired infection than in health care-associated infection (P = 0.084), whereas *Enterococcus* species and *S*. *aureus* were more likely to be isolated in health care-associated infection (P = 0.001 and P = 0.028, respectively).

**Table 2:** Pathogens isolated from 180 patients with community-onset complicated intraabdominal infection within 48 hours of surgery or intervention.

Pathogen isolated	Total,	Community-acquired infection,	Health care-associated infection,
	n = 223 (%)	n = 171 (%)	n = 52 (%)
*Escherichia coli*	94 (42.2)	76 (44.4)	18 (34.6)
Viridans streptococcus and β-hemolytic streptococcus	34 (15.2)	30 (17.5)	4 (7.7)
*Enterococcus* species^a^	26(11.7)	13 (7.6)	13 (25.0)
*Klebsiella pneumoniae*	18 (8.1)	13 (8.2)	5 (9.6)
*Citrobacter* species	10 (4.5)	7 (4.1)	3 (5.8)
*Enterobacter* species	9 (4.0)	7 (4.1)	2 (3.8)
*Pseudomonas aeruginosa*	7 (3.1)	6 (3.5)	1 (1.9)
*Staphylococcus aureus* ^a^	6 (2.7)	2 (1.2)	4 (7.7)
*Candida* species	5 (2.2)	4 (2.3)	1 (1.9)
*Proteus* species	5 (2.2)	5 (2.9)	-
*Klebsiella oxytoca*	2 (0.9)	1 (0.6)	1 (1.9)
*Bacteroides fragilis*	3 (1.3)	3 (1.8)	-
*Serratia* species	1 (0.4)	1 (0.6)	-
Other	3 (1.3)	3 (1.8)	-

^a^ *P* value <0.05 between the two groups.

The extended-spectrum cephalosporin resistance rate among *Enterobacteriaceae* isolates causing community-acquired infection was 6% (7/111) and that among *Enterobacteriaceae* isolates causing health care-associated infection was 21% (6/29). Carbapenem resistant *Enterobacteriaceae* strain was not isolated. Eighty-five percent (29/34) of *Streptococcus* species isolates were susceptible to penicillin, and 85% (22/26) of *Enterococcus* species isolates were susceptible to ampicillin.

### Initial antibiotic therapy

The most commonly used empirical antibiotic regimen was third generation cephalosporin plus metronidazole (52%) (Table 3). The pattern of initial antibiotic therapy was similar between patients with unsuccessful and successful initial antibiotic therapy.

**Table 3:** Initial antibiotic regimen in 514 patients with community-onset complicated intraabdominal infection.

Initial antibiotic regimen	Total,	Initial antibiotic therapy	
	n = 514 (%)	Failure,	Success,
		n = 48 (%)	n = 466 (%)
Monotherapy	112 (21.7)	9 (18.8)	103 (22.1)
1^st^ g. cephalosporin	2 (0.4)	1 (2.1)	1 (0.2)
2^nd^ g. cephalosporin or cephamycin	24 (4.7)	-	24 (5.2)
3^rd^ g. cephalosporin	50 (9.7)	4 (8.3)	46 (9.9)
β-lactam/β-lactamase inhibitor	10 (1.9)	-	10 (2.1)
Fluoroquinolone	9 (1.7)	-	9 (1.9)
Carbapenem	11 (2.1)	3 (6.3)	8 (1.7)
Metronidazole	6 (1.2)	1 (2.1)	5 (1.1)
Combination therapy	402 (78.3)	39 (81.3)	363 (77.9)
2^nd^ g. cephalosporin or cephamycin + metronidazole	76 (14.8)	3 (6.3)	73 (15.7)
3^rd^ g. cephalosporin + metronidazole	266 (51.8)	26 (54.2)	240 (51.5)
Fluoroquinolone + metronidazole	25 (4.9)	4 (8.3)	21 (4.5)
β-lactam/β-lactamase inhibitor + metronidazole	7 (1.4)	1 (2.1)	6 (1.3)
3^rd^ g. cephalosporin + metronidazole + aminoglycoside	13(2.5)	-	13 (2.8)
Carbapenem + glycopeptide	4 (0.8)	1 (2.1)	3 (0.6)
Other^a^	11 (2.1)	4 (8.3)	7 (1.5)

g., generation.

^a^ Each “other” regimen was used in fewer than 1% of patients.

### Outcomes and costs

Patients with unsuccessful initial antibiotic therapy had poor clinical outcomes and incurred higher medical costs, compared with patients with successful initial antibiotic therapy (Table 4). Failure of initial antibiotic therapy was significantly associated with increased mortality. Using multiple linear regression analysis, we found that health care-associated infection, steroid use, neurologic disease or malignancy as a comorbid condition, generalized peritonitis, ICU care, and Acute Physiology and Chronic Heath Evaluation (APACHE) II score ≥13 were significantly related to the number of parenteral antibiotic days. Similarly, we found that health care-associated infection, steroid use, malignancy, generalized peritonitis, ICU care, and APACHE II ≥13 were significantly related to hospital length of stay and medical costs. After adjustment for these covariates, patients with unsuccessful initial therapy received an additional 2.9 days of parenteral antibiotic therapy (95% confidence interval [CI], 0.8–4.9 days), were hospitalized for an additional 5.3 days (95% CI, 2.0−8.5 days), and incurred $3,287 (US dollars) in additional inpatient charges (95% CI, $2,070−$4,505) (Table 5).

**Table 4:** Outcomes of 514 patients with community-onset complicated intraabdominal infection stratified by failure or success of initial antibiotic therapy.

Outcome and cost	Total,	Initial antibiotic therapy		*P* value
	n = 514	Failure,	Success,	
		n = 48	n = 466	
Development of tertiary peritonitis (%)	14 (2.7)	5 (10.4)	9 (1.9)	0.006
In-hospital mortality (%)	16 (3.1)	6 (12.5)	10 (2.1)	0.002
Parenteral antibiotic days, median (IQR)	8 (6–12)	14.5 (8–23.5)	7 (6–11)	<0.001
Hospital length of stay, median days (IQR)	9 (7–15)	15 (10–27.5)	9 (7–14)	<0.001
Overall medical costs, median $ (US dollars, IQR)	3805 (2640−6306)	6981 (3964−15528)	3707 (2617−5641)	<0.001

**Table 5:** Covariate adjusted means of parenteral antibiotic days, length of hospital stay, and overall costs of hospitalization stratified by failure or success of initial antibiotic therapy.

	Initial antibiotic therapy		Difference	95% CI of difference
	Failure, n = 48	Success, n = 466		
Parenteral antibiotic days	13.5	10.6	2.9	0.8–4.9
Length of hospital stay, days	18.9	13.7	5.3	2.0−8.5
Overall costs, $ (US dollars)	9,176	5,889	3,287	2,070−4,505

CI, confidence interval.

### Risk factors for failure of initial antibiotic therapy

Among significant univariate variables with *P* values <0.05 in Table 1, age, ICU care, health care-associated infection, heart disease, neurologic disease, solid cancer, complicated appendicitis, perforation of intestine, and APACHE II ≥13 were included in the multiple logistic regression model. Independent risk factors associated with failure of initial antibiotic therapy were health care-associated infection (adjusted odds ratio [aOR], 9.95; 95% CI, 5.09−19.4; P <0.001), solid cancer (aOR, 2.96; 95% CI, 1.12−7.84; P = 0.029), and APACHE II ≥13 (aOR, 2.34; 95% CI, 1.16−4.70; P = 0.017).

## Discussion

We found that 9% of patients admitted from the community with cIAI experienced failure of initial antibiotic therapy in South Korea. Failure of initial antibiotic therapy was associated with poor clinical outcomes and increased costs. After adjustment for covariates, failure of initial antibiotic therapy was significantly associated with longer duration of antibiotic therapy (2.9 additional days), increased length of hospital stay (5.3 additional days), and higher medical costs ($3,287 additional). Independent risk factors for the failure of initial antibiotic therapy were health care-associated infection, solid cancer, and APACHE II ≥13.

In the previous studies with a similar operational definition of the failure of initial antibiotic therapy in cIAIs, the failure rate of initial antibiotic therapy was 21–27% [5,6]. In contrast, the failure rate in the present study was only 9%. The relatively low failure rate may be probably because most patients in the present study were treated with broad-spectrum cephalosporin based regimen whereas most patients in the previous studies received a β-lactam/β-lactamase inhibitor such as amoxicillin/clavulanate. However, a direct comparison of the effect of the initial antibiotic regimen between studies performed in different countries and settings has no clinical meaning. Clinical outcome, cost-effectiveness, and effect on antibiotic resistance of empirical broad-spectrum cephalosporin versus empirical β-lactam/β-lactamase inhibitor for cIAIs need to be further evaluated in South Korea.

There are a few studies examining the impact of initial antibiotic therapy for cIAIs on outcomes and medical costs [5–8]. Inappropriate initial antibiotic therapy or failure of initial therapy was associated with poor outcomes and increased costs. However, in these studies, the epidemiological category of infection (community-acquired versus health care-associated) was not assessed, or only patients with community-acquired cIAI were enrolled. In the present study, we enrolled all patients with community-onset cIAI encompassing health care-associated infection and found that failure of initial antibiotic therapy adversely affected mortality and medical costs. In addition, we found that health care-associated infection was an independent risk factor for the failure of initial antibiotic therapy and increased length of stay and costs in community-onset cIAIs. Since community-onset cIAIs usually require an urgent or emergent operation, an assessment of health care-associated risk factors may frequently be overlooked, and community-onset health care-associated cIAIs may be regarded and treated as community-acquired cIAIs. Antimicrobial-resistant pathogens are more common in health care-associated cIAI than in community-acquired cIAI [11]. Therefore, to reduce failure of initial antibiotic therapy and improve outcomes and costs in patients admitted from the community with cIAI, it is important to evaluate health care-associated risk factors, select an appropriate antibiotic regimen accordingly, and perform early and adequate source control.

In the practice guidelines for cIAIs, empirical anti-enterococcal therapy is recommended for patients with health care-associated infection, particularly those with postoperative infection, those who have previously received antibiotics selecting for *Enterococcus* species, and immunocompromised patients [4,12–15]. Empirical anti-MRSA therapy is also recommended for patients with health care-associated infection who are known to be colonized with MRSA or at risk of having MRSA infection due to prior significant antibiotic exposure [4]. In the present study, *Enterococcus* species and MRSA accounted for about 33% of community-onset health care-associated cIAIs and were significantly more likely to be isolated in community-onset health care-associated cIAIs than in community-acquired cIAIs. Therefore, anti-enterococcal and anti-MRSA coverage should be considered in patients with severe community-onset heath care-associated cIAI to prevent failure of initial antibiotic therapy.

Our study has several limitations. Microbial pathogens were isolated in only 35% of patients with cIAI, and most infections were monomicrobial. Therefore, the analysis of the appropriateness of selected initial antibiotics against isolated pathogens was difficult in the present study, because this could be done in a limited number of patients and was not thought to be reliable enough. In the real clinical situation, many surgeons do not routinely obtain operative specimens for bacterial culture in cIAIs. Also in the practice guidelines for cIAIs, routine cultures from lower-risk patients with community-acquired cIAI are considered optional [4,16,17]. Therefore, to suggest the recommendation of appropriate empirical antibiotic regimens for community-onset cIAIs in South Korea, microbiological and susceptibility data obtained from routine cultures of operative specimens in prospective studies are required. Recently, Blot et al. proposed an alternative classification of intra-abdominal infections for selecting adequate empirical antibiotics [18]. This classification has a grid based on anatomical disruption, severity of disease, and either site of acquisition and/or recent antibiotic exposure. This grid should be validated in these future prospective studies. In addition, in contrast with previous studies [5,8,19], anaerobes were isolated in a small proportion of patients in the present study. This was probably because of inadequate anaerobic culture rather than different epidemiology. For adequate anaerobic culture results, rapid submission of specimens to the laboratory is required [20]. However, in most hospitals in South Korea, most operative or drainage specimens tend not to be delivered within the appropriate time frame to the laboratory, and anaerobic cultures are usually not performed in most drainage specimens. In a future prospective study, these problems pertaining to anaerobic culture should be considered and resolved.

In conclusion, failure of initial antibiotic therapy in community-onset cIAIs was significantly associated with longer duration of antibiotic therapy, increased length of hospital stay, and higher medical costs, even after adjustment for potential confounders. Independent risk factors for failure of initial antibiotic therapy were health care-associated infection, solid cancer, and APACHE II ≥13. Therefore, to improve outcomes and costs in patients with community-onset cIAI, rapid assessment of health care-associated risk factors and severity of disease, selection of an appropriate antibiotic regimen accordingly, and early infection source control should be performed.

## Supporting Information

S1 Dataset This spreadsheets file contains the raw data in our analyses. (XLSX)
